# Randomized controlled trial of oatmeal consumption versus noodle consumption on blood lipids of urban Chinese adults with hypercholesterolemia

**DOI:** 10.1186/1475-2891-11-54

**Published:** 2012-08-06

**Authors:** Jian Zhang, Lixiang Li, Pengkun Song, Chunrong Wang, Qingqing Man, Liping Meng, Jenny Cai, Anne Kurilich

**Affiliations:** 1 Chinese Center for Disease Control and Prevention, Beijing 100050, China; 2 PepsiCo China Foods, 168 Xizhangmiddle Road, Shanghai, 200001, China; 3 Long Term Research, PepsiCo Inc, 617w.main St, Barrington, IL, 60010, USA

**Keywords:** Oats, Oatmeal, Cholesterol, Chinese adults

## Abstract

**Background:**

Cardiovascular disease (CVD) is the leading cause of death in China and worldwide. Whole grain oats can reduce risk of CVD by reducing total and LDL-cholesterol, major risk factors for CVD. While this association has been established in many populations, data from Asian populations is limited. Thus, this study investigated the impact of oat consumption on cholesterol levels in Chinese adults. Male and female data from this work were previously published separately in mandarin in two Chinese journals. The combined male and female data were reanalyzed and are presented here.

**Methods:**

A randomized, controlled, parallel-arm study was conducted at Beijing Hospital, Beijing china. Subjects were adults (men and women) with mild to moderate hypercholesterolemia. The oat group (n=85) consumed 100grams of instant oat cereal versus the control group (n=81) who consumed 100grams of wheat flour-based noodles daily for 6weeks. Laboratory and anthropometric measurements were conducted at baseline and at the end of the 6-week intervention.

**Results:**

Dietary fiber intake increased significantly in the oat group compared to the control group at the end of the 6-week intervention. Total-, LDL-cholesterol and waist circumference decreased significantly in the oat group compared to the control. HDL-cholesterol decreased significantly in the control group versus the oat group. There were no significant changes in blood pressure, other anthropometric or laboratory measures between the two groups at the end of the intervention.

**Conclusions:**

Instant oatmeal consumed daily for 6 weeks significantly increased fiber intake and decreased major risk factors for CVD in Chinese adults with hypercholesterolemia. Increased consumption of whole grains, including oats, should continue to be encouraged.

## Background

Cardiovascular disease (CVD) is the leading cause of death in China [1] as well as worldwide [2]. Established risk factors for CVD include elevated total cholesterol (TC), elevated low density lipoprotein cholesterol (LDL-C), low high density lipoprotein cholesterol (HDL-C) and hypertension. These risk factors are modifiable by lifestyle factors including diet and exercise [3].

Epidemiological studies suggest that diets high in whole grains are associated with a reduced risk of CVD and mortality [4-7]. In China, recent changes to the traditional diet, including significant reductions in wholegrain intake (104g/day in 1982 to 24g/day in 2002) may be contributing to the increased CVD mortality [8].

Wholegrain oats can significantly lower serum TC and LDL-C and reduce risk for CVD [9]. Authoritative regulatory bodies in several countries, including the United States, Canada, Europe and Malaysia, have reviewed the available evidence and allow a health claim on food labels regarding the relationship between oat consumption and a reduction in blood cholesterol concentration and reduced risk of CVD [10-13].

Data regarding impact of oat consumption on CVD risk factors in Asian populations, including the Chinese population, is limited. Therefore, the present study investigated the relationship between instant oatmeal intake and CVD risk factors in a sample of urban Chinese adults with mild to moderate hypercholesterolemia.

## Methods

### Study design

This was a randomized, controlled, parallel-arm study conducted at Beijing Hospital, Beijing China. Screening of potential study subjects was held in August and September 2008. The six week intervention was conducted from October 2008 through December 2008 during which the subjects visited the research site once per week. The study protocol was approved by the ethics committee of the Institute of Nutrition and Food Safety, Chinese center for Disease control and Prevention. Participants provided written informed consent before any study procedures were performed. Due to the nature of the food products serving as control (wheat noodles) and test (oatmeal) product, it was not possible to blind the subjects or the researchers, however, the statistician analyzing the data was blinded to the treatment groups.

### Study participants

Based on the diagnostic standards in the *Chinese Guidelines on Prevention and Treatment of Dyslipidemia in Adults*[14] the selection criteria were as follows: (1)males and females 35 to 70 years of age; (2) serum total cholesterol ≥5.18mM, low density lipoprotein cholesterol ≥3.37mM; (3) no serious liver, kidney, digestive tract disease, diabetes or other metabolic diseases; (4) no use of cholesterol lowering medication or foods that have been approved by the China State Food and Drug Administration as functional foods with blood lipid lowering properties in the last 6 months; and (5) did not habitually consume oat products. Screening of study participants was done in August and September 2008. Based on data obtained from a basic health questionnaire, food frequency questionnaire and results of a fasting blood lipid test, 182 subjects (109 females, 73 males) were selected to participate and randomized to either the oat group or the control group. Participants were instructed to consume their normal diet during the study period. Participants who smoked could not have plans to change smoking habits during the treatment period.

### Diet and study product

A 3-day 24-hour dietary recall was conducted at the start and end of the study to determine food and nutrient intake. The 24 hour dietary recall questionnaire was validated in the 2002 Chinese Nutrition and Health Survey project. SAS 8.2 software was used to calculate nutrient intake based on Chinese Food composition Table.

Prior to beginning the intervention, all participants were provided with basic nutrition counseling following the Chinese Food Pagoda guidelines which consisted of advice to consume a variety of foods including cereals, fruits, vegetables, dairy and lean meats and to reduce sodium and limit alcohol intake. Participants were instructed not to change their exercise habits and to maintain their normal dietary habits with the exception of replacing a portion of a staple food product (rice, steamed bread, noodles, etc.) with the intervention product (oats or wheat noodles). Oatmeal (Quaker Instant Oatmeal produced by PepsiCo Foods (China) co., Ltd.) was packaged in 200g (dry wt) packages and 4 packages were provided to participants one week followed by 3 packages the next week. Participants receiving the oatmeal were instructed to prepare ½ a package (~100g dry wt, providing approximately 3.6g soluble fiber) each day according to the on-pack preparation instructions (with hot water or milk). Consumption of the prepared amount could be split across two or three meals each day for the six week intervention period. Wheat noodles (“Shengchu” brand processed wheat flour based noodles) were purchased in 450g packages at the local grocery store and distributed to participants on a weekly basis. Participants were instructed to prepare and consume 100g (dry wt) each day. All subjects in the control group were shown a 100g portion of dry noodles that had been weighed on a scale to help them visualize the amount that they should prepare and consume each day. Wheat based noodles are a standard staple in the diets of this population and this amount can easily be consumed in one meal. Each participant was asked to record the quantity of the test food they consumed each day. The oat and noodle packs were distributed once each week to facilitate weekly communication with the participants and discuss intake of food and any relevant concerns or issues linked with the daily oat or noodle consumption.

### Anthropometric and laboratory measurements

Anthropometric measurements were conducted at baseline and at the end of the six week intervention period. Standing height was measured. Participant weight was measured while wearing light weight clothing and without shoes. Waist circumference was measured on a horizontal plane at the level of the iliac crest using a non-stretch anthropometric tape. Two consecutive readings of blood pressure were taken according to methods described in the 2010 Chinese Guidelines for the management of hypertension [15] with the subject in a seated position after 5 minutes of rest. The mean of the two measurements was used for statistical analysis. Fasting blood samples were collected at baseline and at the end of the intervention period. Blood biochemical parameters were measured using a Shimazu 7600 fully automatic biochemical analyzer and included serum total cholesterol (TC), serum triglycerides (TG), high density lipoprotein cholesterol (HDL-C), low density lipoprotein cholesterol (LDL-C), apolipoprotein A1 (ApoA1), apolipoprotein B (ApoB) and plasma glucose.

### Statistical methods

The statistician was blinded to the treatment groups. All statistical summaries and analyses were performed using SAS V 8.2 for windows (SAS Institute, cary, NC). Categorical data were summarized as frequencies and proportions within each group. When analyzing for group differences among the categories, a 2-sided Fisher’s exact test was used. Effect of intervention on dietary composition was analyzed as change from baseline to minimize the bias of any baseline differences. Baseline data and changes from baseline were analyzed using f-test from analysis of covariance (ANCOVA) where the group effect was fixed and the covariates were age, sex, smoking status, alcohol consumption, educational status, and medication use. The effects of intervention on anthropometrics, blood pressure, lipids and glucose were also analyzed for baseline group differences and as change from baseline after six weeks. Summaries of these outcomes were presented as least squares means (LSM) and standard errors (SE) obtained from the ANCOVA model to account for any baseline unbalance in the groups and the effects of the covariates. Statistical significance was set at P<0.05.

## Results

### Study compliance

During the study period there were 9 subjects from the control group and 7 subjects from the oat group that dropped out or were eliminated due to non-compliance with the study protocol. Two subjects from each group were eliminated due to incorrect recording of food amounts as determined by unreasonably low calculated daily calorie intake values (< 500 kcal / day),while 2 subjects from the control group and 1 subject from the oat group were eliminated due to use of products that may reduce serum cholesterol. In addition, 5 subjects from control group and 4 subjects from the oat group dropped out due to personal reasons such as losing interest in the study or travel requirements. A total of 85 subjects in the oat group and 81 subjects in the control group were included in the final data analysis. There were no adverse events reported during the intervention period except for some complaints about the taste of the oat product.

Baseline characteristics of the two test groups are provided in Table 1. There were no statistically significant differences between groups for any of the demographic characteristics or any of the anthropometric measures. There was a statistical difference between groups in ApoB levels at baseline with the oat group having slightly higher levels than the control group (p=0.044). However, both groups were within the normal reference range of 0.60-1.94g/L.

**Table 1:** Baseline characteristics of subjects

**Characteristic**	**Oat group (n=85)**	**Control group (n=81)**	**P Between groups**
**Age (y)**^**1**^	52.7 (0.69)	53.7 (0.73)	0.341
**Male (%)**^**2**^	38.8%	39.5%	>0.999
**Height (cm)**^**3**^	165.1 (0.49)	165.3 (0.50)	0.788
**Waist circumference (cm)**^**3**^	86.7 (0.92)	86.9 (0.94)	0.887
**Weight (kg)**^**3**^	69.7(0.94)	69.8 (0.97)	0.948
**Body mass Index**^**3**^	25.5 (0.32)	25.5 (0.33)	0.964
**Smoker (%)**^**1**^	18.8%	13.6%	0.405
**Alcohol Use (%)**^**1**^	32.9%	29.6%	0.738
**Medication Use (%)**^**1**^	50.6%	56.8%	0.441
**Systolic BP (mmHg)**^**3**^	124.7 (1.74)	129.0 (1.78)	0.085
**Diastolic BP**^**3**^	80.3 (1.06)	79.7 (1.09)	0.689
**Total cholesterol (mmol/L)**^**3**^	6.26 (0.074)	6.09 (0.076)	0.129
**Triglycerides (mmol/L)**^**3**^	2.06 (0.103)	1.89 (0.106)	0.279
**High Density Lipoprotein (mmol/L)**^**3**^	1.47 (0.029)	1.51 (0.030)	0.337
**Low Density Lipoprotein (mmol/L)**^**3**^	4.30 (0.075)	4.17 (0.077)	0.237
**Plasmaglucose (mmol/L)**^**3**^	5.63 (0.097)	5.47 (0.099)	0.277
**Apo A1 (g/L)**^**3**^	1.59 (0.032)	1.57 (0.033)	0.554
**Apo B (g/L)**^**3**^	0.96 (0.017)	0.91 (0.018)	0.044

^1^LSM (SE) and p-value obtained from ANOVA.

^2^Proportion and p-value obtained from 2-sided Fisher’s Exact test.

^3^LSM (SE) and p-value obtained from ANCOVA, where covariates were age, sex, smoking status, alcohol consumption, education status, medication use.

Covariates were evaluated in the analysis of baseline characteristics to determine their effect on each measure prior to intervention. Age was a significant covariate in systolic blood pressure and blood glucose. Sex was a significant covariate in height, waist circumference, weight, systolic blood pressure, diastolic blood pressure, total cholesterol, triglycerides, HDL-C and ApoA1 levels. Smoking status was a significant covariate in total cholesterol, LDL-C and ApoB levels. Alcohol consumption was a significant covariate in height and diastolic blood pressure. Education status was a significant covariate in waist circumference, BMI and systolic blood pressure. Medication use was not a significant covariate for any characteristics. Due to the mixed and overlapping influences of these covariates, all covariates were kept in the subsequent analyses of dietary compositions and changes in anthropometric and lipid measures after 6 weeks of intervention.

### Dietary intake

Dietary compositions at baseline and after 6 weeks of intervention are presented in Table 2. Baseline dietary composition was consistent between the groups for all components except percent of energy from dietary protein which was significantly higher among the oat group than the control group (p=0.004). After 6 weeks of intervention, dietary fiber significantly increased among the oat group compared to the control group (p<0.001). No other changes in composition were notable between the groups.

**Table 2:** Diet compositions

**Diet characteristic**	**LSM (SE)**^**1**^		
	**Oat group (n=85)**	**Control group (n=81)**	**P Between groups**
**Energy (MJ/d) change from Baseline**	**0.30 (0.193)**	**0.32 (0.198)**	**0.942**
**Baseline**	7.6 (0.20)	7.7 (0.20)	0.687
**Week 6**	7.9 (0.20)	8.1 (0.20)	
**Protein (g/d)change from Baseline**	**4.6 (1.97)**	**1.7 (2.02)**	**0.315**
**Baseline**	64.8 (1.83)	61.1 (1.88)	0.168
**Week 6**	69.3 (1.82)	62.8 (1.86)	
**Protein (% energy) change from Baseline**	**0.45 (0.309)**	**-0.14 (0.317)**	**0.191**
**Baseline**	14.3 (0.24)	13.3 (0.25)	0.004
**Week 6**	14.8 (0.28)	13.1 (0.29)	
**Fat (g/d)change from Baseline**	**2.9 (2.58)**	**1.2 (2.64)**	**0.634**
**Baseline**	62.5 (2.77)	61.7 (2.84)	0.841
**Week 6**	65.5 (2.52)	62.9 (2.59)	
**Fat (% energy) change from Baseline**	**0.36 (0.817)**	**-0.58 (0.837)**	**0.428**
**Baseline**	30.3 (0.86)	29.4 (0.88)	0.472
**Week 6**	30.6 (0.77)	28.8 (0.79)	
**Cholesterol (mg/d) change from Baseline**	**-39.7 (21.73)**	**-38.5 (22.27)**	**0.968**
**Baseline**	312 (19.1)	292 (19.5)	0.460
**Week 6**	272 (18.1)	253 (18.5)	
**SFA (% energy) change from Baseline**	**-0.66 (0.261)**	**-0.34 (0.267)**	**0.389**
**Baseline**	8.7 (0.27)	7.9 (0.27)	0.051
**Week 6**	8.0 (0.24)	7.6 (0.25)	
**MUFA (% energy) change from Baseline**	**0.21 (0.414)**	**-0.52 (0.425)**	**0.218**
**Baseline**	12.6 (0.41)	12.3 (0.42)	0.602
**Week 6**	12.9 (0.39)	11.8 (0.40)	
**PUFA (% energy) change from Baseline**	**0.38 (0.388)**	**0.28 (0.398)**	**0.868**
**Baseline**	7.9 (0.39)	8.2 (0.40)	0.632
**Week 6**	8.3 (0.34)	8.5 (0.35)	
**Carbohydrate (g/d) change from Baseline**	**14.2 (7.12)**	**16.5 (7.30)**	**0.825**
**Baseline**	247 (6.4)	259 (6.6)	0.173
**Week 6**	260 (7.2)	276 (7.4)	
**Carbohydrate (% energy) change from Baseline**	**0.41 (0.859)**	**1.08 (0.881)**	**0.588**
**Baseline**	54.9 (0.92)	56.6 (0.94)	0.197
**Week 6**	55.3 (0.83)	57.7 (0.85)	
**Dietary fiber (g/d) change from Baseline**	**7.1 (0.60)**	**1.4 (0.62)**	**<0.001**
**Baseline**	12.2 (0.52)	11.4 (0.53)	0.296
**Week 6**	19.3 (0.55)	12.9 (0.56)	

^**1**^ LSM (SE) and p-value obtained from ANCOVA,where covariates were age, sex, smoking status, alcohol consumption, education status, medication use.

### Anthropometric measures and blood pressure

Anthropometric measures and blood pressure after 6 weeks and as change from baseline are presented in Table 3. Waist circumference significantly decreased after 6weeks in the oat group compared to the control group (p=0.002). No other measures changed significantly.

**Table 3:** Changes in anthropometric measures and blood pressure during study period

**Measures**	**LSM (SE)**^**1**^		
	**Oat group (n=85)**	**Control group (n=81)**	**P Between groups**
**Waist circumference (cm) change from Baseline**	**−1.27 (0.473)**	**0.85 (0.485)**	**0.002**
**Baseline**	86.7 (0.92)	86.9 (0.94)	0.887
**Week 6**	85.4 (0.82)	87.7 (0.84)	
**Weight (kg) change from Baseline**	**0.46 (0.306)**	**0.67 (0.313)**	**0.621**
**Baseline**	69.7(0.94)	69.8 (0.97)	.0948
**Week 6**	69.8 (0.93)	70.4 (0.96)	
**Body mass Index change from Baseline**	**0.04 (0.092)**	**0.25 (0.094)**	**0.108**
**Baseline**	25.5 (0.32)	25.5 (0.33)	0.964
**Week 6**	25.6 (0.32)	25.7 (0.32)	
**Systolic Blood Pressure change from Baseline**	**1.01 (1.120)**	**0.85 (1.477)**	**0.919**
**Baseline**	124.7 (1.74)	129.0 (1.78)	0.085
**Week 6**	125.7 (1.65)	129.9 (1.69)	
**Diastolic Blood Pressure change from Baseline**	**−0.13 (0.916)**	**0.76 (0.938)**	**0.500**
**Baseline**	80.3 (1.06)	79.7 (1.09)	0.689
**Week 6**	80.1 (0.97)	80.4 (1.00)	

^**1**^ LSM (SE) and p-value obtained from ANCOVA, where covariates were age, sex, smoking status, alcohol consumption, education status, medication use.

### Serum lipids and plasma glucose

**Figure 1:**
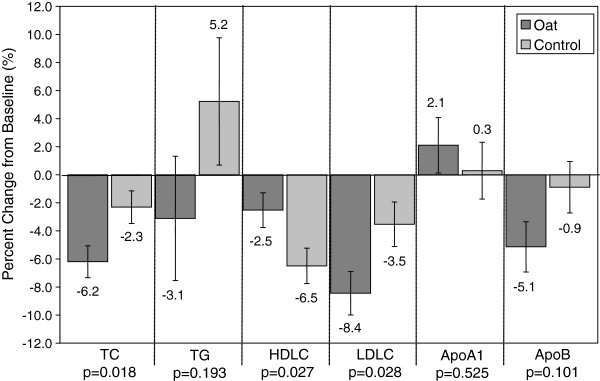
Percentage of change in lipids.

Serum lipids and plasma glucose after 6weeks and as change from baseline are presented in Table 4. The oat group showed significant decreases in total cholesterol (p=0.015) and LDL-C (p=0.028) compared to the control group. In addition, HDL-C decreased significantly in the control group compared to the oat group (p=0.017). Effects of intervention were observed for these same lipids when summarized as a percentage change from baseline (Figure 1). There were no significant differences in plasma glucose levels between the two groups.

**Table 4:** Changes in lipids and glucose during study period

**Measures**	**LSM (SE)**^**1**^		
	**Oat group (n=85)**	**Control group (n=81)**	**P Between groups**
**Total cholesterol (mmol/L) change from Baseline**	**−0.41 (0.071)**	**−0.15 (0.073)**	**0.015**
**Baseline**	6.26 (0.074)	6.09 (0.076)	0.129
**Week 6**	5.85 (0.085)	5.94 (0.087)	
**Triglycerides (mmol/L) change from Baseline**	**−0.15 (0.089)**	**−0.04 (0.091)**	**0.404**
**Baseline**	2.06 (0.103)	1.89 (0.106)	0.279
**Week 6**	1.91 (0.107)	1.85 (0.110)	
**HDLC (mmol/L) change from Baseline**	**−0.04 (0.018)**	**−0.10 (0.018)**	**0.017**
**Baseline**	1.47 (0.029)	1.51 (0.030)	0.337
**Week 6**	1.43 (0.032)	1.41 (0.033)	
**LDLC (mmol/L) change from Baseline**	**−0.39 (0.067)**	**−0.17 (0.069)**	**0.028**
**Baseline**	4.30 (0.075)	4.17 (0.077)	0.237
**Week 6**	3.91 (0.081)	4.00 (0.083)	
**Apo A1 (g/L) change from Baseline**	**0.02 (0.29)**	**−0.02 (0.029)**	**0.432**
**Baseline**	1.59 (0.032)	1.57 (0.033)	0.554
**Week 6**	1.61 (0.035)	1.55 (0.036)	
**Apo B (g/L) change from Baseline**	**−0.06 (0.016)**	**−0.02 (0.017)**	**0.102**
**Baseline**	0.96 (0.017)	0.91 (0.018)	0.044
**Week 6**	0.91 (0.017)	0.89 (0.017)	
**Plasma glucose (mmol/L) change from Baseline**	**−0.30 (0.057)**	**−0.17 (0.059)**	**0.118**
**Baseline**	5.63 (0.097)	5.47 (0.099)	0.277
**Week 6**	5.34 (0.088)	5.29 (0.090)	

^**1**^ LSM (SE) and p-value obtained from ANCOVA, where covariates were age, sex, smoking status, alcohol consumption, education status, medication use.

## Discussion

The results of this study show that when instant oats are consumed daily in place of another staple food (rice, steamed bread, noodles, etc.) compared to wheat noodles as replacement for another staple food, there is a significant increase in dietary fiber intake and significant decreases in waist circumference, TC and LDL-C in Chinese adults with moderate hypercholesterolemia. During the intervention period there was a 6.2% decrease in TC for the oat group compared to a 2.3% decrease in the control group. There was also an 8.4% decrease in LDL-C for the oat group compared to a 3.5% decrease in the control group.

The oat group consumed 100 grams of instant oats per day which provided ~3.6 grams of soluble fiber. Oat products containing β-glucan soluble fiber decrease TC and LDL-C by altering bile acid metabolism and increasing bile acid excretion [16,17]. The reductions in TC and LDL-C observed in this study are similar to previous reports in free living individuals. Van Horn et al. [18] reported a 5.2% reduction in TC in healthy adults consuming 60g/day of either oat bran or oatmeal for 6weeks as part of a low fat diet. Karmally and colleagues [19] provided 3g/day of β-glucan from ready-to-eat (RTE) oat cereal for 6weeks to adults with mild to moderate hypercholesterolemia and observed reductions of 4.5% in TC and 5.3% in LDL-C. In a recent trial a 5.4% reduction in TC along with an 8.7% decrease in LDL-C was reported following consumption of 3g/day of β-glucan from ready-to-eat (RTE) oat cereal for 12 weeks in overweight or obese adults [20]. A 2007 cochrane review reported results of a meta-analysis based on 8 randomized clinical trials that used oats as a wholegrain intervention. A significant effect of oat consumption to lower both TC (P=.0005) and LDL-C concentrations (P=.0008) was observed. The mean percentage reduction in LDL-C from baseline (95% confidence interval) was 4.9% (7.6% to 2.4%) [9].

The prevalence of dyslipidemia in Chinese adults has been reported to be 2.9% for hypercholesterolemia (serum TC ≥5.18 mmol/L) and 11.9% for hypertriglyceridemia (serum TG ≥1.70 mmol/L) based on a nationally representative sample of subjects [21]. However, certain subgroups of the population have a higher reported prevalence of hypercholesterolemia. The prevalence is higher in adults over 60 years of age and has been reported to be 20.2% in males and 38.7% in females in this age group [22]. The prevalence of dyslipidemia has also been reported to be higher for urban (21%) compared to rural (17.7%) Chinese adults [21].The reduction in LDL-C observed in this study is of the magnitude to reduce risk of CHD since every 1% reduction in LDL-C is associated with a decreased risk for CHD of 1% to 3% [23,24].

A dietary pattern characterized by a high intake of vegetables, fruit and soy is one protective lifestyle factor associated with a marked decreased risk of coronary heart disease, cerebrovascular disease, and overall CVD mortality in Chinese men and women [25].The traditional Chinese diet included a high intake of vegetables and coarse grains, which are the main sources of total and insoluble dietary fiber in the Chinese population [8,26]. However significant dietary changes have occurred in recent years which may be contributing to the prevalence of hypercholesterolemia within the population. The national average daily intake of cereals decreased from 510g/day in 1982 to 402g/day in 2002. The amount of coarse grains decreased from 104g/day to 24g/day in the same time period [8]. Average intake of total dietary fiber decreased from 22.6g/day in 1989 to 18.1g/day in 2006 and insoluble dietary fiber intake decreased from 15.1g/day in 1989 to 11.9g/day in 2006 [26]. The 2007 Chinese food based dietary guidelines (FBDGs) issued by the Chinese Nutrition Society include a recommendation to include an appropriate amount of coarse grains. The proposed guidelines recommend a coarse grain intake, including whole grains, of no less than 50 grams per day for adults [8]. In this study the oat group had a significant increase in dietary fiber intake (7.1 grams/day) compared to the control group (1.4grams/day, p<0.001) during the treatment period. Intake of course grains was not calculated in this study.

The control group had a significant decrease in HDL-C concentration compared to the oat group during the intervention period (p=0.017). Dietary factors that impact HDL-C concentration include total fat and *trans* fat intakes along with the ratio of dietary saturated to unsaturated fat. The decrease in HDL-C in this study is puzzling since there were no significant changes in energy or total, saturated, mono- or poly-unsaturated fat intakes during the intervention period. There also was no significant change in ApoA-1 observed during the treatment period in this study. ApoA-1 concentration is also influenced by the ratio of polyunsaturated to saturated fat [27].

Waist circumference decreased 1.27 cm in the oat group compared to a 0.85 cm increase in the control group (p=0.002). Abdominal obesity is strongly associated with metabolic disturbances such as hypertriglyceridemia and insulin resistance [28]. Consumption of whole grains has been associated with smaller waist circumference in population studies [29,30]. Maki et al [20] reported a ~1.5cm reduction in waist circumference in overweight adults consuming 3g/day of β-glucan for 12weeks from RTE oat cereal. The clinical implications of a reduction in waist circumference without simultaneous changes in BMI or weight should be investigated.

There was no significant effect of oat consumption on blood pressure at the end of the 6 week treatment period in this study. Previous studies have reported mixed results of the effect of oats, β-glucan or whole grains on blood pressure. Keenan et al [31] reported reduced systolic and diastolic blood pressure in a pilot trial with oats. Whole-grain diets reduced blood pressure in mildly hypercholelsterolemic men and women [32]. Other investigators reported no effect on blood pressure after consumption of foods containing oat β-glucan [33], oats [34] and RTE oat cereal [20]. One confounding factor in this study is that slightly more than 50% of the total subjects were using medications including antihypertensive medication during the intervention.

One limitation of this study is exercise/activity levels were not reported however the subjects were instructed not to make any changes to their habitual diet or other lifestyle factors during the intervention. Another limitation is that antihypertensive medications were allowed during the trial and therefore the impact of the oat intervention on blood pressure was confounded. As the soluble fiber content of the diet before and after intervention could not be calculated, it is not possible to determine if there were changes to soluble dietary fiber intake over the course of the study.

## Conclusions

Consumption of 100 grams of instant oatmeal per day significantly reduced TC, LDL-C and waist circumference in moderately hypercholesterolemic Chinese men and women. Replacement of a staple food with oatmeal significantly increased dietary fiber intake. Recent Chinese dietary guidelines include a recommendation to consume an appropriate amount of coarse grains including whole grains. The results of this study demonstrate that consumption of a wholegrain oat cereal has beneficial effects on risk factors for CVD.

## Abbreviations

CVD: Cardiovascular Disease; TC, Total cholesterol; LDL-C: Low Density Lipoprotein cholesterol; HDL-C: High Density Lipoprotein cholesterol; TG: Triglycerides; ApoA1: Apolipoprotein A1; ApoB: Apolipoprotein B; ANCOVA: Analysis of covariance; LSM: Least Square mean; SE: Standard Error; RTE: Ready To Eat; FBDGs: Food Based Dietary Guidelines.

## Competing interests

Authors AK and JC are employees of PepsiCo, Inc. and Pepsi Co china Foods, respectively.

Authors JZ, LL, PS, QM, LM and CW are employees of Institute of Nutrition and Food Safety, Chinese center for Disease control and Prevention, and declare no conflicts of interests.

## Authors’ contributions

The study was designed by JZ. LL, QM and CW carried out the study and collected the data (under the supervision of JZ). PS was responsible for the dietary survey and nutrient calculations. LM and AK were responsible for data analyses. JZ and AK were responsible for interpretation of results and manuscript writing. JC, AK and JZ reviewed manuscript drafts and the final manuscript. All authors read and approved the final manuscript.

## Author details

^1^Chinese Center for Disease Control and Prevention, Beijing 100050, China.^2^PepsiCo China Foods, 168 Xizhangmiddle Road, Shanghai 200001, China.^3^Long Term Research, PepsiCo Inc, 617w.main St, Barrington, IL 60010, USA.
